# “The dream is that there’s one place you go”: a qualitative study of women’s experiences seeking care from Long COVID clinics in the USA

**DOI:** 10.1186/s12916-024-03465-1

**Published:** 2024-06-13

**Authors:** Linnea I. Laestadius, Jeanine P. D. Guidry, Megan M. Wahl, Paul B. Perrin, Kellie E. Carlyle, Xiaobei Dong, Raouf Gharbo, Celeste Campos-Castillo

**Affiliations:** 1 Zilber College of Public Health, University of Wisconsin-Milwaukee, Milwaukee, WI USA; 2 Department of Communication and Cognition, Tilburg University, Tilburg, The Netherlands; 3 School of Data Science and Department of Psychology, University of Virginia, Charlottesville, VA USA; 4 School of Population Health, Virginia Commonwealth University, Richmond, VA USA; 5 Department of Physical Medicine and Rehabilitation, Virginia Commonwealth University School of Medicine, Richmond, VA USA; 6 Department of Media and Information, Michigan State University, East Lansing, MI USA

**Keywords:** Long COVID, Women, Qualitative, Post COVID, Access to care, Quality of care

## Abstract

**Background:**

Seeking and obtaining effective health care for Long COVID remains a challenge in the USA. Women have particularly been impacted, as they are both at higher risk of developing Long COVID and of facing gendered barriers to having symptoms acknowledged. Long COVID clinics, which provide multidisciplinary and coordinated care, have emerged as a potential solution. To date, however, there has been little examination of U.S. patient experiences with Long COVID clinics and how patients may or may not have come to access care at a Long COVID clinic.

**Methods:**

We conducted semi-structured interviews with 30 U.S. women aged 18 or older who had experienced Long COVID symptoms for at least 3 months, who had not been hospitalized for acute COVID-19, and who had seen at least one medical provider about their symptoms. Participants were asked about experiences seeking medical care for Long COVID. Long COVID clinic-related responses were analyzed using qualitative framework analysis to identify key themes in experiences with Long COVID clinics.

**Results:**

Of the 30 women, 43.3% (*n* = 13) had been seen at a Long COVID clinic or by a provider affiliated with a Long COVID clinic and 30.0% (*n* = 9) had explored or attempted to see a Long COVID clinic but had not been seen at time of interview. Participants expressed five key themes concerning their experiences with seeking care from Long COVID clinics: (1) Access to clinics remains an issue, (2) Clinics are not a one stop shop, (3) Not all clinic providers have sufficient Long COVID knowledge, (4) Clinics can offer validation and care, and (5) Treatment options are critical and urgent.

**Conclusions:**

While the potential for Long COVID clinics is significant, findings indicate that ongoing barriers to care and challenges related to quality and coordination of care hamper that potential and contribute to distress among women seeking Long COVID care. Since Long COVID clinics are uniquely positioned and framed as being the place to go to manage complex symptoms, it is critical to patient wellbeing that they be properly resourced to provide a level of care that complies with emerging best practices.

## Background

In addition to posing significant acute health risks, COVID-19 has been linked to the condition known as Long COVID or post-acute sequelae of COVID-19 (PASC) [1]. The prevalence of Long COVID is notably high [2, 3], with recent estimates between 11 and 15% among U.S. adults who tested positive for COVID-19 [4, 5]. Women are at higher risk of Long COVID symptoms than men [3, 4, 6], with data from the U.S. Household Pulse Survey (HPS) indicating that 13.8% of women who ever had COVID-19 were actively experiencing Long COVID symptoms in January 2024 [7]. Long COVID can cause respiratory, vascular, neurological, digestive, and immune symptoms, among others [8–10]. Those with Long COVID report low health-related quality of life. High rates of missed working days due to fatigue, and significant limitations to their daily activities [5, 11, 12]. Despite the significant nature of Long COVID symptoms, seeking and obtaining effective health care has been a frequent challenge and source of distress [13–16].

Long COVID clinics, which provide multidisciplinary and coordinated care for those with Long COVID [17], have emerged as a potential solution given the multiple organ symptoms impacted [18–20]. Healthcare providers who themselves have been diagnosed with Long COVID have also stressed the importance of a “one-stop shop” for Long COVID [21]. To date, however, there has been little examination of U.S. patient experiences with Long COVID clinics and how patients may or may not have come to access care at a Long COVID clinic. In September 2023, the U.S. Department of Health and Human Services allocated nine grant awards to expand multidisciplinary Long COVID clinics, particularly in underserved areas [17]. More research is needed to understand patient preferences and experiences for accessing Long COVID clinics to better inform Long COVID clinic procedures and practices.

While scant, there is some research on patient experiences with Long COVID clinics, often arising in explorations of barriers to Long COVID care more broadly. A study about COVID-19 and mental health [13] found that while patients in the UK were broadly aware that such clinics existed, they were confused about the referral process and felt that they lacked clinics in their area. A diary study of twelve patients at a UK Long COVID clinic also found that patients were frustrated by wait times but appreciated referrals for care [22]. Garg et al. [23] administered a survey on perceptions at a single Long COVID clinic in the USA in 2021, finding that while most patients were satisfied and appreciated having their experiences validated, they reported long wait times and travel distances as well as scheduling and financial barriers to seeing clinic-referred specialists. German Long COVID patients have reported both a lack of clinics and bureaucratic barriers to being seeing by Long COVID clinics [24]. Clinic leads report that a lack of established treatment protocols and insufficient staffing present barriers to care [25].

Understanding obstacles and limitations such as these is critical, not just in light of increased federal funding for Long COVID clinics, but also because Long COVID clinics have a tremendous potential for meeting patient needs by providing multidisciplinary and integrated care in a single location [21, 26]. The importance of specialty clinics, despite current limitations, must also be considered within the context of a health care system in which only 28% of U.S. physicians felt very or somewhat confident treating patients with Long COVID in 2022 [27]. When new evidence-based guidelines and treatments become available, Long COVID clinics will be particularly well positioned to implement them. Further research is thus needed to understand current experiences with U.S. Long COVID clinics and how they could be strengthened, particularly for women as they are both at higher risk of Long COVID and at higher risk of facing gendered barriers to having symptoms taken seriously by medical providers [3, 28, 29]. Therefore, this study conducted semi-structured interviews to examine women’s experiences seeking care from U.S. Long COVID clinics and how they may or may not have successfully accessed such care.

## Methods

To be eligible, participants had to identify as a woman aged 18 or older who resided in the USA, had experienced Long COVID symptoms at the time of recruitment for at least 3 months, had not been hospitalized for acute COVID-19, and had seen at least one medical provider about their Long COVID symptoms.

We recruited participants between January 2023 and May 2023 from emails collected in a prior online panel survey study about Long COVID care experiences, email outreach through the team’s professional and personal networks, and unpaid social media posts on Twitter (now known as X), Reddit (r/womenshealth, r/samplesize, r/COVIDsupport), Instagram, Mastodon, and Facebook (“COVID 19 research involvement group”). Social media posts and emails linked to an online survey we created using the Qualtrics survey platform to screen for eligibility, gather demographic data, and collect contact information. We also screened IP addresses to verify U.S. status. Making use of the screener data, we adopted a maximum variation sampling approach [30] in which we selected potential participants with the aim of capturing a diverse pool of experiences, enrolling women from a range of ages, racial/ethnic groups, education levels, and insurance types. Institutional Review Board approval was received from the University of Wisconsin—Milwaukee.

We selected a target sample size of 30 participants, which is slightly larger than what prior studies suggest is needed to reach saturation [31, 32]. We opted for a larger sample size to account for the potential heterogeneity of experiences accessing and obtaining care from Long COVID clinics, which impacts the “information power” of the sample and necessitates a larger sample size [33]. Four participants came from the prior Long COVID survey, with the rest from social media and professional/personal network outreach efforts by team members. Participants who completed interviews received a $50 gift card. Participants were from 16 different U.S. states and ranged in age from 19 to 67. See Table 1 for participant demographics.

**Table 1:** Summary participant characteristics

**Characteristics**	*N* = 30	%
**Age**		
Teens	1	3.3%
20s	4	13.3%
30s	7	23.3%
40s	11	36.7%
50s	5	16.7%
60s	2	6.7%
**Race/Ethnicity**		
White non-Hispanic	17	56.7%
Black	4	13.3%
Black/White	2	6.7%
Hispanic	1	3.3%
Hispanic/White	1	3.3%
Asian/White	3	10.0%
Middle Eastern/White	2	6.7%
**Level of Educational Attainment**		
High school or GED	4	13.3%
Four Year College Degree	15	50.0%
Graduate Degree	11	36.7%
**U.S. Census Region**		
West	6	20.0%
South	5	16.7%
Midwest	9	30.0%
Northeast	9	30.0%
Pacific	1	3.3%
**Insurance Type**		
Private (employer, exchange)	19	63.3%
Medicaid	7	23.3%
Medicare	4	13.3%
**Long COVID Clinic Status**		
Visited Long COVID Clinic	13	43.3%
Explored or Attempted Visiting Long COVID Clinic	9	30.0%
Did not Explore/Did not Mention	8	26.7%

Of the 30 women, 43.3% (*n* = 13) mentioned having been seen at a Long COVID clinic or by a provider described as being affiliated with a Long COVID clinic (virtually or in person), 30.0% (*n* = 9) explored or attempted to be seen at a Long COVID clinic but had not been seen at time of interview, and 26.7% (*n* = 8) had not explored Long COVID clinics, were unaware of them, or did not mention them. See Table 2 for additional details by participant.

**Table 2:** List of participant demographics

Participant Number	Race/Ethnicity	Age	Region	Education	Insurance Type	COVID Clinic Status
1	White/Black/American Indian	40 s	Midwest	Bachelors	Medicare	No/Not aware
2	White	30 s	Midwest	High School	Medicaid	No/Not aware
3	Black	20 s	Midwest	Graduate	Private	No/Not mentioned
4	White	40 s	Northeast	Bachelors	Private	Explored or Attempted
5	White/Asian	40 s	Midwest	Graduate	Private	Yes
6	White	40 s	West	Graduate	Private	Yes
7	Black	60 s	West	Bachelors	Medicare	Explored or Attempted
8	White	40 s	South	Graduate	Private	Explored or Attempted
9	White	20 s	Northeast	Bachelors	Private	Yes
10	Black	30 s	Midwest	Graduate	Private	Yes
11	White	30 s	West	Graduate	Medicaid	Explored or Attempted
12	White	40 s	Midwest	High School	Private	Yes
13	White	40 s	Northeast	Bachelors	Medicaid	Yes
14	White	30 s	West	Graduate	Private	Explored or Attempted
15	White	60 s	South	Bachelors	Medicare	No/Not mentioned
16	White	20 s	Midwest	Bachelors	Private	No/Not mentioned
17	White	40 s	Pacific	Graduate	Medicaid	Yes
18	White	50 s	Northeast	Bachelors	Medicaid	No/Not mentioned
19	White/Middle Eastern	40 s	Northeast	Graduate	Private	Yes
20	White/Black	30 s	Northeast	Bachelors	Medicaid	Yes
21	Hispanic	Teens	South	High School	Medicaid	No/Not mentioned
22	White	50 s	West	Graduate	Private	Yes
23	White/Asian	40 s	Northeast	Bachelors	Private	No/Not mentioned
24	White/Middle Eastern	50 s	Northeast	Graduate	Private	Explored or Attempted
25	White	50 s	Northeast	High School	Private	Yes
26	White	50 s	Midwest	Bachelors	Medicare	Explored or Attempted
27	White/Asian	40 s	South	Bachelors	Private	Yes
28	White	30 s	Midwest	Bachelors	Private	Explored or Attempted
29	White/Hispanic	20 s	West	Bachelors	Private	Explored or Attempted
30	Black	30 s	South	Bachelors	Private	Yes

After providing verbal consent, participants completed semi-structured interviews via Zoom video, which were then auto-transcribed by Zoom and manually checked for accuracy. Interviews lasted on average 65 min. As part of a larger study about women and Long COVID, interview questions considered experiences seeking medical care for Long COVID, the types of medical providers participants had seen, barriers to obtaining care, and participant suggestions for improving Long COVID care. Long COVID clinic discussions developed organically as participants recounted their full care seeking journeys. Following each interview, we prepared brief reflexive memos capturing key insights and reflections on the interview process.

We analyzed interview data using a framework analysis approach [34]. The lead author and two trained students independently open coded the first five transcripts using MAXQDA 2022. We discussed initial coding and developed a codebook. The lead author applied this codebook for coding the remaining transcripts, with the opportunity for new codes to be added via inductive coding. We found that no new substantive dimensions of Long COVID clinic access or quality of care were identified as coding of the final transcripts occurred, indicating that data saturation was reached [31]. Following framework analysis steps, the lead author then charted Long COVID clinic codes for each participant, with summaries and illustrative quotes, into a MAXQDA matrix table to facilitate interpretation. The full team reviewed theme names and descriptions before findings were finalized.

## Results

**Fig. 1:**
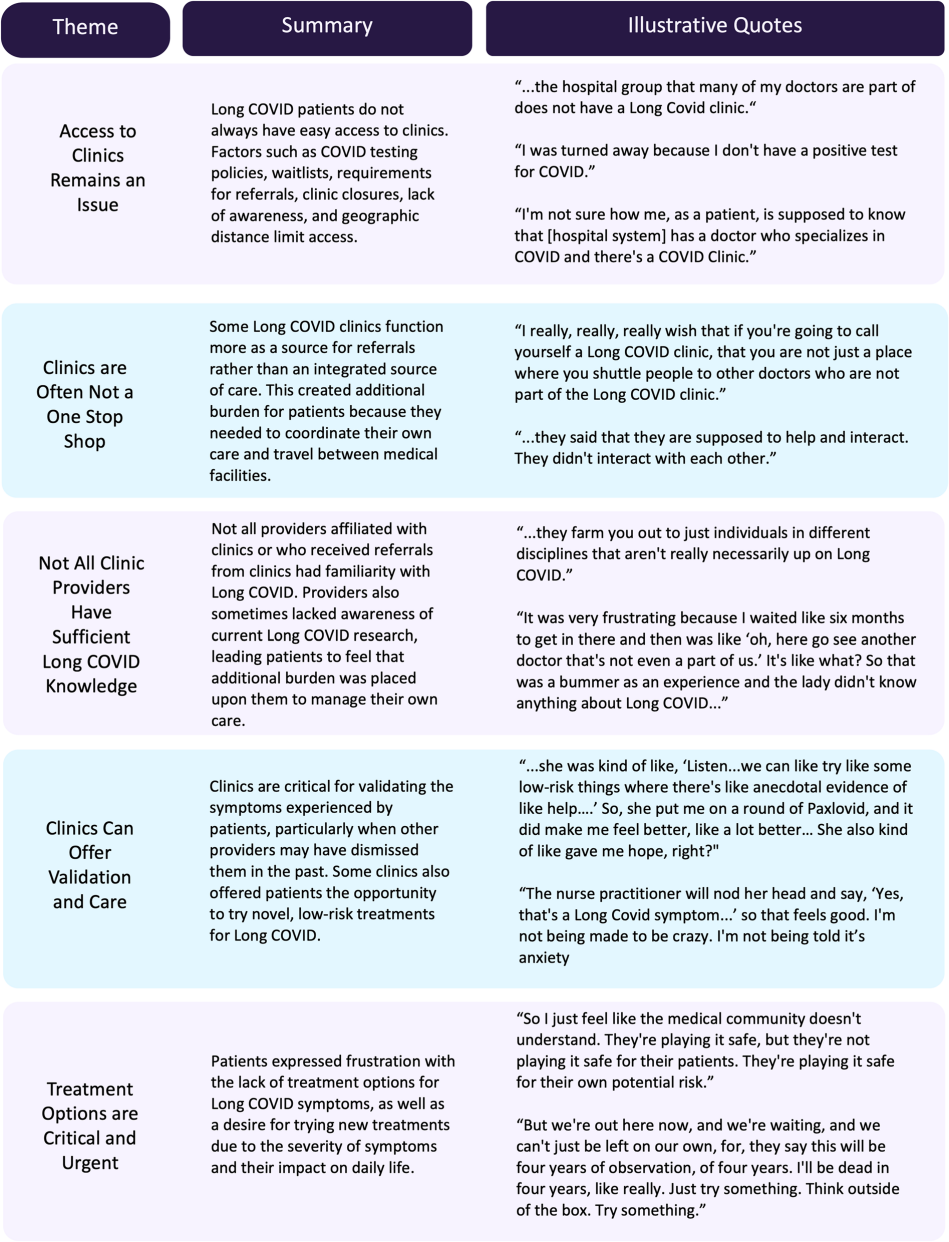
Long COVID clinic care themes and quotes

Qualitative analysis generated five overarching themes related to experiences with Long COVID clinics: (1) Access to clinics remains an issue, (2) Clinics are not a one stop shop, (3) Not all clinic providers have sufficient Long COVID knowledge, (4) Clinics can offer validation and care, and (5) Treatment options are critical and urgent. Participant quotes below are identified by participant number, age group, and U.S. Census region. We removed names of specific medical centers and providers to ensure confidentiality. See Fig. 1 for a summary of each theme.

### Access to clinics remains an issue

Participants who had been seen, or attempted to be seen, at a Long COVID clinic reported multiple barriers to access, including awareness, referrals, and clinic location and organization. Many participants described how they had started their care journey with primary care providers (PCPs) or emergency departments. Two participants, both in the Midwest, indicated that their PCPs had never mentioned Long COVID clinics to them, but that they were now interested in attending one after learning about them through probing by the interviewer. For example, one of the two indicated that she was happy with her PCP, but would prefer to see a specialty clinic due to additional insight they could likely offer:

Participant: My doctor’s been pretty good as far as working with me and keeping me up to date and stuff so. Even my fiancée, he’s like, I think he fractured like a rib from coughing so hard. Yeah, so it’s like, yeah, we need... doctors specifically for that, you know? Like, you can go to like a COVID clinic, you know, or something*.*

Interviewer: … I think that there’s been a little bit in the news about Long COVID clinics. Is that something that you’ve looked into at all, or that your doctors ever mentioned?

Participant: Uum, no, no. I was just thinking about it. Like, that would actually be a good idea to have something like that for people like my fiancé, who don't have a doctor who can help them out. But I would probably be interested in it, too, because, just because it’s, they’re more, that’s their specialty. You know, I would, I would check that out, too.” (P1, 40s, Midwest)

Another participant expressed a lack of knowledge about the existence of clinics but explained that she had eventually been directed to a Long COVID clinic affiliated provider after being trapped in a cycle of emergency department admissions for her symptoms. She noted that she did not know how others would find out that such a service was available.

“I’m not sure how me, as a patient, is supposed to know that [hospital system] has a doctor who specializes in COVID and there’s a COVID Clinic... like I’m just not sure how patients are supposed to figure that out. And maybe I’m just not very savvy, but I just didn’t see clear signs. Like, I think, kind of like how you, you know, if I break my arm, I need to go see an orthopedic type thing." (P30, 30s, South)

Thus, lack of awareness about clinics and providers who fail to suggest clinics as an option contribute to delays in accessing care and, in some cases, can prevent patients from ever connecting with a Long COVID clinic. For others, the obstacle was not knowledge about clinics, but rather that their PCP was actively discouraging or blocking their attempt to be seen at a clinic. When Long COVID clinics or insurance required referrals, this obstacle was heightened. One participant who had still not been to a Long COVID clinic due to this barrier explained how her PCP and the need for a referral had prevented her from being seen at the clinic she hoped to attend:

“Every single time I’ve asked [my PCP] about if I should see like a Long COVID specialist, or if we should take that into consideration, into her writing the referral, and maybe they would want to manage things better than how she has been, she’s kind of written me off even. She told me at our last visit… that the Long COVID clinics would want to see that I had gone to a cardiologist before accepting me anyways. Which I’m not sure is true, but I, I looked up the one at [X university hospital]… and I tried to explain that it doesn’t look like that at all, because they have the specialists there. But she still didn’t want to fill out the paperwork, so I kind of got stuck with that.” (P29, 20s, West)

Another barrier came in the form of clinics requiring a positive polymerase chain reaction (PCR) or antibody test for COVID-19 to be seen. This was a particular issue for those who had become ill during the first wave of COVID when testing was not widely available. While some participants indicated they had initially been turned away from a clinic but persisted until they found one that did not require a positive test, others still had not seen a clinic due to this barrier. One woman explained, “My primary care provider suggested that I, like, be referred to a Long COVID Clinic. It is nearby. But I was turned away because I don’t have a positive test for COVID” (P14, 30s, West). The lack of a positive test had initially led another participant to attend a clinic that was a 6-hour round-trip from her home (P22, 50s, West). She stated she was relieved when she contracted COVID a second time because she could finally obtain a positive test to get access to a Long COVID clinic closer to her home.

Finally, logistics and organizational challenges posed barriers to accessing clinics. As mentioned above, location was sometimes a challenge. Another participant noted that she had explored the idea of a Long COVID clinic, but that,

“…the hospital group that many of my doctors are part of does not have a Long COVID clinic or specialist group that could act as a clearing house for all this stuff, or I could go there and do it all at once.” (P8, 40s, South)

Others experienced long wait times to be seen at clinics, including one participant who had waited for months for an appointment only to be told that the “clinic had actually been shut down, because the one nurse who was running it left” (P20, 30 s, Northeast). Another participant described feeling discouraged when a visit was rescheduled without notice:

“I was supposed to have an appointment in early March with another place. So, I had booked in December, and when I showed up, they were like, ‘Oh, we changed your appointment to the end of May and didn't tell you.’ And I was like, ‘Okay, F you.’ So, I just canceled that. I was like, ‘I’m not going to go here.’” (P5, 40s, Midwest)

These quotes highlight the logistical struggles of successfully being seen at clinics. Participants also stressed that navigating these struggles was further complicated by the taxing nature of their Long COVID symptoms.

### Clinics are often not a one stop shop

A common frustration was that the Long COVID clinic did not operate a singular, coordinated clinic that was prepared to meet the breadth of Long COVID health needs. Several participants expressed disappointment about the clinic structures they experienced once they were able to be seen.

“I really, really, really wish that if you're going to call yourself aLong COVID clinic, that you are not just a place where you shuttle people to other doctors who are not part of the Long COVID clinic. I don't understand why they set up, ‘COVID clinics’that are basically places that do that. And that seems to me the majority of them.” (P19, 40s, Northeast)

For some, Long COVID clinics were a single telehealth nurse who provided guidance and referrals. While referrals themselves were not inherently a negative experience and participants were generally pleased to see specialists, the lack of coordination between providers and the lack of a centralized location for care created additional burdens, forcing women to schedule additional appointments, serve as a go-between for providers, and travel between appointments. The lack of coordination was also sometimes seen as limiting the quality of care received, with a sense that clinics did not see the full picture of symptoms they were experiencing.

“The dream is that there’s like one place you go, and they do all your imaging, all your blood tests and all your follow up, and they coordinate all the care between all the specialists, and they just like, take care of you. And also, like that, you can coordinate, like having fewer, having to go fewer places, fewer different times. Not having to go to one place for this, to another place for that.” (P13, 40s, Northeast)

“That whole Long, Long Haul COVID Clinic, they said that they are supposed to help and interact. They didn’t interact with each other. Like I had to bring all my research to people myself and it’s, and when you’re already tired and sick, it’s really hard to do that. Like I really feel like with Long COVID, there needs to be some type of advocates that are assigned to each people, especially people with like lower resources. I’ve been living off my savings. It’s now gone since I got sick over two years ago. I’ve already been denied disability.” (P17, 50s, Pacific)

The quotes above capture participant desire for an integrated one-stop shop model where multiple tests can be done in the same visit and where providers confer about results and treatments plans. The second quote also indicates the need for navigators to assist patients in handling the complexities of care across multiple providers and facilities, as well as frustration about the gap between the ideal clinic model and actual lived experience of attending a clinic. Between the logistical barriers to being seen at clinics, and the logistical challenges of navigating care once at a clinic, participants described a clinic system that often relies on patients serving as their own advocates and care managers despite grappling with symptoms such as fatigue and brain fog.

### Not all clinic providers have sufficient long COVID knowledge

Because of the referral-oriented nature of the clinics, the experiences that participants had with Long COVID clinics depended heavily on the quality of the specialists to whom they were referred. Even within clinics, different specialists had offered varying levels of awareness about Long COVID. Participants recognized that current medical knowledge about Long COVID was limited, but felt that some of the providers they had seen via referrals lacked the baseline level of knowledge that they expected for clinic affiliation.

“I went here thinking that you send me to people who basically see Long COVID patients! That’s what the name implies, and I didn’t expect you all to be experts in it, and nobody is. But at least if you’re seeing Long COVID patients day in, day out, you have more experience than the average person. It, it was very frustrating because I waited like six months to get in there and then it was like ‘oh, here go see another doctor that’s not even a part of us.’It’s like what? So that was a bummer as an experience and the lady didn't know anything about Long COVID and was one of the people that was very like ‘maybe you’re just not taking deep enough breaths.’ It’s like, what? Thank you. That’s super helpful [sarcasm].” (P19, 50s, Northeast)

One participant noted that one of her Long COVID clinic providers had admitted limited knowledge about Long COVID and that the clinic primarily had represented the financial interests of the hospital system: “My [specialist], sweet as she is, looked me in the eye and said, ‘I don’t know what I’m doing. They haven't educated me about Long COVID at all. There’s an administrator that thinks we can make money off of this, and that’s why I’m seeing all these people.’ She’s like,‘I hate it. I don't have any help.’”(P6, 40s, West). While the provider had still been able to be supportive by offering additional testing, the participant stressed the need for better provider education. Further, the concern about health systems profiting off Long COVID patients by adding clinics without sufficient expertise to support them is notable given that concerns about financial exploitation have, to date, primarily focused on alternative providers offering unfounded treatments [35].

There was a strong desire that Long COVID clinic providers be more informed about current developments in Long COVID: “…one centralized place where everyone knows some, where everyone knows what’s going on, like the latest, would be more helpful” (P5, 40s, Midwest). A participant who had attended both conventional Long COVID clinics and a private telehealth Long COVID clinic described significant differences between the two, with the conventional clinic far behind in terms of both awareness of current developments and collaboration:

“There [at the telehealth Long COVID clinic], when I read an article and send it to them, they’ve already read it, even if it’s something that just came out…and they’re willing to try things, whereas with you know [the hospital Long COVID clinic] they farm you out to just individuals in different disciplines that aren’t really necessarily up on Long COVID... They didn’t function as an integral whole. It was, it was kind of a carve up the elephant, you know, and the Long COVID doctors theoretically should have had knowledge and interest in root causes, and then meeting together with the other doctors, and, you know, like sharing information. Or I feel like these doctors should all get together once a week, once a month, whatever, and, and go through,what’s the latest? What are the latest articles that have hit?” (P22, 50s, West)

### Clinics can offer validation and care

Despite the concerns raised above, several of the participants who had attended Long COVID clinics were relieved to finally have had their experiences with Long COVID validated and had found at least one clinic affiliated provider that had run diagnostic tests or offered suggestions for symptom relief. While validation alone is not sufficient to relieve symptoms, legitimation of symptoms and status as a Long COVID patient was described as a pivotal and cathartic experience. As indicated in the quotes below, this appreciation was in part grounded in feelings of having been dismissed or gaslit by primary or emergency care providers, and a sense of relief at not being treated like they were “crazy”:

“If I didn’t have the Covid clinic, I don’t know where I’d be…I can remember that email exchange when I described myself to him, and I’m just sitting there waiting, and he got back to me. 'Yes, absolutely. You come here', and I just burst into tears…you recognize this is a pattern, and I’m part of it. Oh, thank you. Oh, my God, you know too! And what a miserable thing to be happy about!” (P25, 50s, Northeast)

“The nurse practitioner will nod her head and say, ‘Yes, that’s a Long COVID symptom…there’s nothing that’s outlandish to us. There’s nothing that we haven’t heard. You’re welcome to tell, you know. Tell us everything. There’s nothing that surprises us....’ so that feels good. I’m not being made to be crazy. I’m not being told it’s anxiety....” (P10, 30s, Midwest)

While less common than the aforementioned emotional support, some participants noted that their clinic providers had also offered them access to off-label prescriptions that gave symptom relief they have been unable to obtain elsewhere. As expressed in the quote below, clinic willingness to try different solutions also provided a sense of hope:

“Definitely the, the Long COVID clinic has helped... like she actually, um... she put me on some courses of Paxlovid, you know, and she was kind of like, ‘Listen, there’s no like studies blah blah blah, or like big randomized trials like that. But, like we can like try like some low-risk things where there’s like anecdotal evidence of like help….’ So, she put me on a round of Paxlovid, and it did make me feel better, like a lot better… She also kind of like gave me hope, right?” (P5, 40s, Midwest)

### Treatment options are critical and urgent

As suggested in the prior theme, participants particularly valued Long COVID clinic providers who were willing to try novel approaches to treat their symptoms. While alternative and complementary treatments, such as mindfulness, heart rate monitors, and acupuncture, were welcomed by some participants, a focus purely on these approaches at times left participants feeling frustrated.

“They think the mindfulness is helpful. It can't hurt to try … It just seems like how many hundreds of dollars was that for you to tell me that. It seems kind of pitifully obvious.” (P25, 50s, Northeast)

“…and literally her recommending like games that I could play on my phone or TV shows for me to watch, to occupy myself. So not really, you know, providing what I would consider medical care.” (P20, 30s, Northeast)

Several participants mentioned that they had experienced benefits from the off-label use of pharmaceutical drugs and that they would welcome low-risk exploratory treatments to see whether they would provide symptom relief. One participant stressed the emotional and physical costs of doing nothing until U.S. Food and Drug Administration (FDA)-approved drugs were available.

“…and there’s the whole RECOVER Study. It’s like, I get it. You want to cross every t and dot every i. But we’re out here now, and we’re waiting, and we can’t just be left on our own, for, they say this will be four years of observation, of four years. I’ll be dead in four years, like really. Just try something. Think outside of the box. Try something.” (P25, 50s, Northeast)

The participant who had signed up for a private telehealth Long COVID clinic expressed similar concerns with waiting until federal approvals for Long COVID treatments has been secured.

“So the doctor at [hospital Long COVID clinic] is kind of poo-pooing the treatments like oh, we only like to do like FDA approved stuff, but it’s like, FDA approved stuff? You know, I’m not doing anything extremely risky. I mean, there’s you know, probably 1 billion people on the planet who are using nicotine every day. Not that it’s good for them, but come on, for me to use a [nicotine] patch, you know, half of the lowest dose patch for 2 weeks? It’s pretty low risk, you know? If I can get some functionality out of that, great. Guanfacine, an ADHD medication. If I can get some improvement out of it. These are the types of things you know, I’m willing to try, and my doctors at [telehealth clinic] are willing to try… So I just feel like the medical community doesn’t understand. They’re playing it safe, but they’re not playing it safe for their patients. They’re playing it safe for their own potential risk.” (P22, 50s, West)

She also noted that the conventional university-affiliated clinics she had visited felt like “palliative care” and that one of her providers there had told her that “you won’t get well here… they’re not really looking at cutting edge treatments” and had provided names that ultimately led her to the telehealth clinic. While the fees associated with the telehealth clinic had posed a significant burden, she felt obligated to continue since the other clinics had largely been unwilling to try new approaches and were dismissive of off-label approaches such as nicotine patches.

## Discussion

Overall, this study’s findings present a picture in which the potential for Long COVID clinics to fill a vital need has been hampered by challenges related to care coordination and quality of care. These findings are consistent with the limited prior work on experiences with Long COVID clinics, particularly regarding the difficulty of being seen at a clinic, frustrations with referrals, and a desire for concrete steps to improve symptoms [13, 23, 24]. The current findings show these key challenges remain 3 years after Long COVID clinics first emerged in 2020 [25]. Barriers created by the lack of a COVID test were particularly concerning given that access to tests was limited during the early phase of the pandemic and that U.S. insurers are no longer required to cover the cost of COVID tests as of May 2023 [36]. Further, not all participants were aware of Long COVID clinics, indicating a continued need for raising public and PCP awareness of Long COVID treatment options. Health department efforts to raise awareness of support and treatment options for those with Long COVID have been relatively limited [37]. Survey research is needed to explore how patients attending Long COVID clinics first learned about their existence and how to become enrolled as a patient.

### Validation and treatment as key functions

We also found that Long COVD clinic experiences were variable and that some women with Long COVID receive more comprehensive, coordinated, and informed care than others. While the current study’s approach does not allow us to generalize concretely based upon effects of insurance type, demographics, or region on these experiences, the variability here suggests that while Long COVID clinics can provide a satisfactory and positive experience even with few formally recognized treatment protocols available, not all clinics are performing optimally. Participants particularly valued clinic providers who heard their concerns, who were informed about the most recent developments with Long COVID diagnostics and treatments, and who were willing to try low risk off-label or alternative treatments while the wait for FDA-approved treatments continues. Many clinics appear to be open to learning about new clinical protocols and joining Long COVID clinic collaboratives [25], which may be a promising approach for ensuring that all clinics benefit from the lessons learned by those who have resources to stay abreast of potential new treatment protocols. Since Long COVID patients have been sharing and crowdsourcing information about their condition on social media [38], online communities may also be a source of valuable information for providers seeking to improve clinic experiences and for patients seeking clinics that align with their needs.

While Long COVID clinics can offer a valuable service even just in validating women’s Long COVID symptoms after they have been “gaslit” by other providers [12, 23], the quality of life and health challenges posed by Long COVID necessitates combining recognition with efforts to relieve symptoms [8, 9, 11]. Providers are faced with navigating the delicate balance between the good that may come from prescribing off-label medications (particularly those that already have been approved for treatment of other illnesses and/or populations) and the principle of “First, do no harm”, especially in the midst and aftermath of a pandemic that seemed to provide more questions than answers [39, 40]. Communication between providers and patients and clear guidance for providers are both of utmost importance. A failure to offer any meaningful guidance for symptom relief can encourage self-treatment, which can also pose health risks and drain financial resources [22, 35].

### Challenges posed by resources, clinical guidance, and coordination of care

On an operational level, health systems seeking to establish Long COVID clinics should reflect on whether they are allocating sufficient resources to ensure that clinics can operate properly, including staffing levels, coordination, and expertise among clinic affiliated providers and specialist referrals. Prior research suggests that clinics need additional resources, including physicians and staff, and that not all clinics are following best practices, specifically for holding interdisciplinary team meetings [25]. The findings here suggest that patients notice when care is uninformed or uncoordinated, and several participants in this study described negative experiences with clinics, contributing to frustration, exhaustion, and at times despair. Poorly implemented and under-resourced Long COVID clinics may contribute to the trauma already experienced by Long COVID patients from their symptoms, social stigma, and medical gaslighting [10]. Since Long COVID clinics are uniquely positioned and framed as being the place to go to handle complex Long COVID symptoms, it is critical to patient wellbeing that they provide some minimum level of care that complies with emerging best practices. Further, for women, who have long been shown to experience increased physical and somatoform symptoms across a host of health conditions [41], lack of validation and attention by health care professionals of Long COVID symptoms may have detrimental effects on future engagement with Long COVID clinics and overall quality of life.

More broadly, our findings highlight two issues that persist in the U.S. health care system and are only compounded by the COVID-19 pandemic. The first issue pertains to fragmented care. In recent years, several innovative delivery models have been initiated to improve care coordination such as Patient-Centered Medical Homes, Accountable Care Organizations (ACOs), and Medicaid Health Homes [42]. However, these models have only been adopted at a limited scale. Moreover, even models such as ACOs have struggled with integrating specialists and coordinating comprehensive patient care plans for complex patients [43, 44]. Our findings highlight the importance of developing and implementing effective strategies for care coordination to provide more effective care to Long COVID patients. Across studies, Long COVID patients consistently voice the need for a multi-disciplinary and integrated approach to their care [21, 22, 24, 45]. One-stop multidisciplinary clinics are critical to “avoid multiple referrals to different specialists,” which would otherwise be needed given the breadth of symptoms experienced by patients, often impacting multiple different organ systems [8, 26]. Improving coordinated care for Long COVID patients may require tying incentives to outcome- and process-based performance. To develop these performance benchmarks, clinical guidelines will be needed. Sound guidelines will necessitate both a large body of clinical research and patient input, specifically from those who hold lived experience with Long COVID [46, 47]. For example, work by groups such as the Patient Led Research Collaborative can offer valuable insight into Long COVID management [48].

The second issue revealed by our study is related to the US public health preparedness. Public health services have historically been underfunded [49, 50]. When hit by the COVID pandemic, the U.S. public health system was ill-equipped to respond efficiently and effectively [50]. One prominent aspect of this failure, as evident in our findings, is lacking sufficient and timely clinical guidance for health care providers. Continued patient experiences with providers who are not fully informed about Long COVID suggest a strong need for additional clinician training related to Long COVID [45].

While challenges with care coordination are common for chronic disease management even in high-income and well-resourced geographic regions [51], the findings in the current study may be even more accentuated in low-income communities within the USA or in medically underserved global regions. Further, if the concerns articulated by this study’s participants are present in relation to Long COVID specialty clinics in the USA, the gaps in care are likely even starker for women in U.S. communities or low-income and middle-income countries with no or less-specialized Long COVID clinics [52]. Finally, while our study focused on women’s experiences due both to their higher risk of developing Long COVID and to gendered barriers to care [2, 28], challenges with limited clinic capacity, coordination of care, and Long COVID expertise among providers likely impact Long COVID patients of all genders. Future research should consider men’s experiences seeking Long COVID care, particularly in light of evidence that symptoms may vary by sex [53, 54].

### Limitations and future directions

Despite the important implications of the current study findings, there are several limitations that should be considered. Participants' self-reported Long COVID status, and symptoms were not independently confirmed. Although many Long COVID symptoms can only be evidenced through self-report, future-related research might consider recruiting participants directly from Long COVID clinics rather than the diverse sampling techniques used in the current study. Additionally, over half of participants identified as White, recruiting and interviews were conducted in English, and education levels were somewhat higher than the U.S. average. Thus, the already notable barriers to obtaining care that were identified may be understated. In the USA, lower-income adults and Black and Hispanic women report higher rates of Long COVID [6, 55]. These women may face heightened barriers to accessing care due to structural and interpersonal racism [14]. Further, everyone in our sample reported having some form of health insurance. Women without health insurance may face additional barriers to receiving referrals or being seen at clinics. These caveats are important for assessing the transferability of findings to other Long COVID populations.

Additionally, while all participants were asked about the care they had sought for Long COVID, not all participants were probed to ensure that a failure to mention Long COVID clinics represented a lack of interest in attending such a clinic. Accordingly, the strong barriers to clinic attendance that we found here may be understated. Finally, Zoom was used for transcription, which heightens the risk of transcription errors. To counteract this, we manually checked transcripts and all quotes were confirmed against the audio for accuracy. Future work should also consider asking participants how they learned about the study to help determine the most effective strategies for recruitment.

## Conclusions

This was the first known study to qualitatively examine the experiences of U.S. women seeking high-quality care from Long COVID clinics. Participants’ experiences converged on the five themes of access to clinics remaining an issue, clinics not being a one stop shop, not all clinic providers having sufficient Long COVID knowledge, the ability of clinics to offer validation and care, and the urgency and critical nature of developing and offering treatment options for Long COVID. These themes provide concrete and actionable suggestions for Long COVID clinics to improve experiences for women experiencing Long COVID symptoms and seeking resources for symptom relief. Incorporating these suggestions will have high potential to improve care experiences for Long COVID and potentially increase overall patient quality of life. Further, Long COVID clinics need additional support in the face of challenges including limited resources and uncertainty about the pandemic and its after-effects, as well as the sheer volume of Long COVID patients as COVID-19 continues to spread. More resources for both research and practice are critically needed to adequately meet the needs of those with Long COVID.

## Data Availability

Transcripts generated and analyzed during the current study are not publicly available to ensure participant confidentiality, but coded segments and codebooks are available from the corresponding author on reasonable request.

## Abbreviations

ACO: Accountable Care Organization
FDA: U.S. Food and Drug Administration
PASC: Post-acute sequelae of COVID-19
PCP: Primary care provider

## References

[1] CDC. Post-COVID Conditions Information for Healthcare Providers. 2023. https://www.cdc.gov/coronavirus/2019-ncov/hcp/clinical-care/post-covid-conditions.html. Accessed 3 Feb 2024.

[2] Nittas V, Gao M, West EA, Ballouz T, Menges D, Hanson SW, et al. Long COVID Through a Public Health Lens: An Umbrella Review. Public Heal Rev. 2022;43:1604501. 10.3389/phrs.2022.1604501. 10.3389/phrs.2022.1604501 PMC8963488 35359614

[3] Chen C, Haupert SR, Zimmermann L, Shi X, Fritsche LG, Mukherjee B. Global Prevalence of Post COVID-19 Condition or Long COVID: A Meta-Analysis and Systematic Review. J Infect Dis. 2022;226(9):1593–607. 10.1093/infdis/jiac136. 35429399 10.1093/infdis/jiac136 PMC9047189

[4] Perlis RH, Santillana M, Ognyanova K, Safarpour A, Trujillo KL, Simonson MD, et al. Prevalence and Correlates of Long COVID Symptoms Among US Adults. JAMA Netw Open. 2022;5:e2238804. 10.1001/jamanetworkopen.2022.38804. 36301542 10.1001/jamanetworkopen.2022.38804 PMC9614581

[5] Ford ND, Slaughter D, Edwards D, Dalton A, Perrine C, Vahratian A, et al. Long COVID and Significant Activity Limitation Among Adults, by Age — United States, June 1–13, 2022, to June 7–19, 2023. Morb Mortal Wkly Rep. 2023;72:866–70. 10.15585/mmwr.mm7232a3. 10.15585/mmwr.mm7232a3 PMC10415000 37561665

[6] Cohen J, van der Meulen Rodgers Y. An intersectional analysis of long COVID prevalence. Int J Equity Heal. 2023;22:261. 10.1186/s12939-023-02072-5. 10.1186/s12939-023-02072-5 PMC10717295 38093291

[7] CDC. Long COVID - Household Pulse Survey. 2024. https://www.cdc.gov/nchs/covid19/pulse/long-covid.htm#print.

[8] Davis HE, McCorkell L, Vogel JM, Topol EJ. Long COVID: major findings, mechanisms and recommendations. Nat Rev Microbiol. 2023;21:133–46. 10.1038/s41579-022-00846-2. 36639608 10.1038/s41579-022-00846-2 PMC9839201

[9] Pinto MD, Downs CA, Huang Y, El-Azab SA, Ramrakhiani NS, Barisano A, et al. A distinct symptom pattern emerges for COVID-19 long-haul: a nationwide study. Sci Rep. 2022;12:15905. 10.1038/s41598-022-20214-7. 36151129 10.1038/s41598-022-20214-7 PMC9508141

[10] Lambert N, Corps S, El-Azab SA, Ramrakhiani NS, Barisano A, Yu L, et al. The other COVID-19 survivors: Timing, duration, and health impact of post-acute sequelae of SARS-CoV-2 infection. J Clin Nurs. 2022. 10.1111/jocn.16541. 36181315 10.1111/jocn.16541 PMC9538514

[11] Walker S, Goodfellow H, Pookarnjanamorakot P, Murray E, Bindman J, Blandford A, et al. Impact of fatigue as the primary determinant of functional limitations among patients with post-COVID-19 syndrome: a cross-sectional observational study. BMJ Open. 2023;13:e069217. 10.1136/bmjopen-2022-069217. 37286327 10.1136/bmjopen-2022-069217 PMC10335413

[12] Owen R, Ashton RE, Skipper L, Phillips BE, Yates J, Thomas C, et al. Long COVID quality of life and healthcare experiences in the UK: a mixed method online survey. Qual Life Res. 2023:1–11. 10.1007/s11136-023-03513-y. 10.1007/s11136-023-03513-y PMC10784347 37740144

[13] Burton A, Aughterson H, Fancourt D, Philip KEJ. Factors shaping the mental health and well-being of people experiencing persistent COVID-19 symptoms or ‘long COVID’: qualitative study. Bjpsych Open. 2022;8:e72. 35307048 10.1192/bjo.2022.38 PMC8987646

[14] Bergmans RS, Chambers-Peeple K, Aboul-Hassan D, Dell’Imperio S, Martin A, Wegryn-Jones R, et al. Opportunities to Improve Long COVID Care: Implications from Semi-structured Interviews with Black Patients. Patient - Patient-centered Outcomes Res. 2022;15:715–28. 10.1007/s40271-022-00594-8. 10.1007/s40271-022-00594-8 PMC9362503 35907120

[15] Russell D, Spence NJ, Chase J-AD, Schwartz T, Tumminello CM, Bouldin E. Support amid uncertainty: Long COVID illness experiences and the role of online communities. SSM - Qual Res Heal. 2022;2:100177. 10.1016/j.ssmqr.2022.100177. 10.1016/j.ssmqr.2022.100177 PMC9531408 36212783

[16] Ladds E, Rushforth A, Wieringa S, Taylor S, Rayner C, Husain L, et al. Persistent symptoms after Covid-19: qualitative study of 114 “long Covid” patients and draft quality principles for services. BMC Health Serv Res. 2020;20:1144. 10.1186/s12913-020-06001-y. 33342437 10.1186/s12913-020-06001-y PMC7750006

[17] Agency for Healthcare Research and Quality. AHRQ Long COVID Care Network. 2024. https://www.ahrq.gov/coronavirus/long-covid/care-network.html. Accessed 3 Feb 2024.

[18] Dean E. What happens inside a long covid clinic? BMJ. 2023;382:p1791. 10.1136/bmj.p1791. 10.1136/bmj.p1791 37678897

[19] Santhosh L, Block B, Kim SY, Raju S, Shah RJ, Thakur N, et al. Rapid Design and Implementation of Post-COVID-19 Clinics. Chest. 2021;160:671–7. 10.1016/j.chest.2021.03.044. 33811910 10.1016/j.chest.2021.03.044 PMC8010340

[20] O’Brien H, Tracey MJ, Ottewill C, O’Brien ME, Morgan RK, Costello RW, et al. An integrated multidisciplinary model of COVID-19 recovery care. Ir J Med Sci. 2021;190:461–8. 10.1007/s11845-020-02354-9. 32894436 10.1007/s11845-020-02354-9 PMC7475726

[21] Ladds E, Rushforth A, Wieringa S, Taylor S, Rayner C, Husain L, et al. Developing services for long COVID: lessons from a study of wounded healers. Clin Med. 2021;21:59–65. 10.7861/clinmed.2020-0962. 10.7861/clinmed.2020-0962 PMC7850205 33479069

[22] Thomas C, Faghy MA, Owen R, Yates J, Ferraro F, Bewick T, et al. Lived experience of patients with Long COVID: a qualitative study in the UK. BMJ Open. 2023;13:e068481. 37185640 10.1136/bmjopen-2022-068481 PMC10151237

[23] Garg A, Subramain M, Barlow PB, Garvin L, Hoth KF, Dukes K, et al. Patient Experiences with a Tertiary Care Post-COVID-19 Clinic. J Patient Exp. 2023;10:23743735231151540. 10.1177/23743735231151539. 10.1177/23743735231151539 PMC9869203 36698619

[24] Schmachtenberg T, Königs G, Dragaqina A, Roder S, Müller F, Müllenmeister C, et al. “There is no one who helps you with it”: experiences of people with long COVID regarding medical care, therapeutic measures, and barriers in the German healthcare system: results of a qualitative study with four focus groups. BMC Heal Serv Res. 2023;23:1160. 10.1186/s12913-023-10170-x. 10.1186/s12913-023-10170-x PMC10601213 37884993

[25] Dundumalla S, Barshikar S, Niehaus WN, Ambrose AF, Kim SY, Abramoff BA. A survey of dedicated PASC clinics: Characteristics, barriers and spirit of collaboration. PMR. 2022;14:348–56. 10.1002/pmrj.12766. 10.1002/pmrj.12766 35038230

[26] Sivan M, Taylor S. NICE guideline on long covid. BMJ. 2020;371:m4938. 33361141 10.1136/bmj.m4938

[27] de Beaumont Foundation. Poll: Doctors Feel Unprepared to Treat Long COVID. 2023. https://debeaumont.org/wp-content/uploads/2023/03/Long-COVID-Brief.pdf.

[28] Au L, Capotescu C, Eyal G, Finestone G. Long covid and medical gaslighting: Dismissal, delayed diagnosis, and deferred treatment. SSM - Qual Res Heal. 2022;2:100167. 10.1016/j.ssmqr.2022.100167 10.1016/j.ssmqr.2022.100167 PMC9448633 36092770

[29] Sebring JCH. Towards a sociological understanding of medical gaslighting in western health care. Sociol Health Ill. 2021;43:1951–64. 10.1111/1467-9566.13367. 10.1111/1467-9566.13367 34432297

[30] Merriam SB, Tisdell EJ. Qualitative Research: A guide to design and implementation. 4th ed. San Francisco: Jossey-Bass; 2016.

[31] Hennink M, Kaiser BN. Sample sizes for saturation in qualitative research: A systematic review of empirical tests. Soc Sci Med. 2022;292: 114523. 10.1016/j.socscimed.2021.114523. 34785096 10.1016/j.socscimed.2021.114523

[32] Guest G, Bunce A, Johnson L. How Many Interviews Are Enough? Field Method. 2006;18:59–82. 10.1177/1525822x05279903. 10.1177/1525822x05279903

[33] Malterud K, Siersma VD, Guassora AD. Sample Size in Qualitative Interview Studies. Qual Health Res. 2016;26:1753–60. 10.1177/1049732315617444. 26613970 10.1177/1049732315617444

[34] Gale NK, Heath G, Cameron E, Rashid S, Redwood S. Using the framework method for the analysis of qualitative data in multi-disciplinary health research. BMC Med Res Methodol. 2013;13:117. 10.1186/1471-2288-13-117. 24047204 10.1186/1471-2288-13-117 PMC3848812

[35] Davies M. Long covid patients travel abroad for expensive and experimental “blood washing.” BMJ. 2022;378: o1671. 10.1136/bmj.o1671. 35820689 10.1136/bmj.o1671

[36] McPhillips D. Free Covid-19 tests aren’t guaranteed after May 11, but there’s still time to stock up. CNN. 2023. https://www.cnn.com/2023/05/01/health/free-covid-tests-phe-wellness/index.html.

[37] Laestadius LI, Guidry JPD, Bishop A, Campos-Castillo C. State Health Department Communication about Long COVID in the United States on Facebook: Risks, Prevention, and Support. Int J Environ Res Pu. 2022;19:5973. 10.3390/ijerph19105973. 10.3390/ijerph19105973 PMC9140570 35627510

[38] Thompson CM, Rhidenour KB, Blackburn KG, Barrett AK, Babu S. Using Crowdsourced Medicine to Manage Uncertainty on Reddit: The Case of COVID-19 Long-haulers. Patient Educ Couns. 2021. 10.1016/j.pec.2021.07.011. 34281723 10.1016/j.pec.2021.07.011 PMC8805953

[39] Coleman DL, Rosoff PM. The Enhanced Danger of Physicians’ Off-Label Prescribing During a Public Health Emergency. J Law Biosci. 2020;7:lsaa031. 10.1093/jlb/lsaa031. 32874596 10.1093/jlb/lsaa031 PMC7453637

[40] Huffman A. The Ethics of Using Off-Label Medications to Treat COVID-19. Ann Emerg Med. 2022;79:A13–5. 10.1016/j.annemergmed.2022.04.007. 10.1016/j.annemergmed.2022.04.007

[41] Kroenke K, Spitzer RL. Gender Differences in the Reporting of Physical and Somatoform Symptoms. Psychosom Med. 1998;60:150–5. 10.1097/00006842-199803000-00006. 9560862 10.1097/00006842-199803000-00006

[42] Landry AY, Erwin CO. Organization of Care. In J. R. Knickman & B. Elbel (Eds.), Health Care Delivery in the United States (12th ed.). 2019. Springer Publishing Company.

[43] Fraze TK, Beidler LB, Briggs ADM, Colla CH. Translating Evidence into Practice: ACOs’ Use of Care Plans for Patients with Complex Health Needs. J Gen Intern Med. 2021;36:147–53. 10.1007/s11606-020-06122-4. 33006083 10.1007/s11606-020-06122-4 PMC7858720

[44] Ganguli I, Lupo C, Mainor AJ, Orav EJ, Blanchfield BB, Lewis VA, et al. Association between specialist compensation and Accountable Care Organization performance. Heal Serv Res. 2020;55:722–8. 10.1111/1475-6773.13323. 10.1111/1475-6773.13323 PMC7518824 32715464

[45] Hawke LD, Nguyen ATP, Sheikhan NY, Strudwick G, Rossell SL, Soklaridis S, et al. Swept under the carpet: a qualitative study of patient perspectives on Long COVID, treatments, services, and mental health. BMC Heal Serv Res. 2023;23:1088. 10.1186/s12913-023-10091-9. 10.1186/s12913-023-10091-9 PMC10568931 37821939

[46] Gorna R, MacDermott N, Rayner C, O’Hara M, Evans S, Agyen L, et al. Long COVID guidelines need to reflect lived experience. Lancet. 2021;397:455–7. 10.1016/s0140-6736(20)32705-7. 33357467 10.1016/s0140-6736(20)32705-7 PMC7755576

[47] Elwyn G, Quinlan C, Mulley A, Agoritsas T, Vandvik PO, Guyatt G. Trustworthy guidelines – excellent; customized care tools – even better. BMC Med. 2015;13:199. 10.1186/s12916-015-0436-y. 26324120 10.1186/s12916-015-0436-y PMC4556022

[48] McCorkell L, Assaf GS, Davis HE, Wei H, Akrami A. Patient-Led Research Collaborative: embedding patients in the Long COVID narrative. Pain Rep. 2021;6:e913. 10.1097/pr9.0000000000000913. 33987484 10.1097/pr9.0000000000000913 PMC8112577

[49] Himmelstein DU, Woolhandler S. Public Health’s Falling Share of US Health Spending. Am J Public Heal. 2016;106:56–7. 10.2105/ajph.2015.302908. 10.2105/ajph.2015.302908 PMC4695931 26562115

[50] Blumenthal D, Fowler EJ, Abrams M, Collins S. Covid-19 — Implications for the Health Care System. N Engl J Med. 2020;383:1698–1698. 10.1056/nejmx200018. 32706956 10.1056/nejmx200018

[51] Fradgley EA, Paul CL, Bryant J. A systematic review of barriers to optimal outpatient specialist services for individuals with prevalent chronic diseases: what are the unique and common barriers experienced by patients in high income countries? Int J Equity Heal. 2015;14:52. 10.1186/s12939-015-0179-6. 10.1186/s12939-015-0179-6 PMC4464126 26051244

[52] Jassat W, Reyes LF, Munblit D, Caoili J, Bozza F, Hashmi M, et al. Long COVID in low-income and middle-income countries: the hidden public health crisis. Lancet. 2023;402:1115–7. 10.1016/s0140-6736(23)01685-9. 37652071 10.1016/s0140-6736(23)01685-9

[53] Khan SA, Ashkar R, Kumari S, Khenhrani RR, Ullah S, Rajpar R, et al. Long COVID syndrome: psychological and sexual dysfunction among survivors of COVID-19 infection. Ann Med Surg. 2023;85:4788–93. 10.1097/ms9.0000000000001153. 10.1097/ms9.0000000000001153 PMC10553038 37811042

[54] Sylvester SV, Rusu R, Chan B, Bellows M, O’Keefe C, Nicholson S. Sex differences in sequelae from COVID-19 infection and in long COVID syndrome: a review. Curr Med Res Opin. 2022;38:1391–9. 10.1080/03007995.2022.2081454. 35726132 10.1080/03007995.2022.2081454

[55] US Census Bureau. Hispanic, Black Adults More Likely to Report Long COVID-19 Symptoms. 2023. https://www.census.gov/library/stories/2023/05/long-covid-19-symptoms-reported.html. Accessed 2 Feb 2024.

