# Correction: “The dream is that there’s one place you go”: a qualitative study of women’s experiences seeking care from Long COVID clinics in the USA

**DOI:** 10.1186/s12916-024-03522-9

**Published:** 2024-07-23

**Authors:** Linnea I. Laestadius, Jeanine P. D. Guidry, Megan M. Wahl, Paul B. Perrin, Kellie E. Carlyle, Xiaobei Dong, Raouf Gharbo, Celeste Campos-Castillo

**Affiliations:** 1 Zilber College of Public Health, University of Wisconsin-Milwaukee, Milwaukee, WI USA; 2 Department of Communication and Cognition, Tilburg University, Tilburg, The Netherlands; 3 School of Data Science and Department of Psychology, University of Virginia, Charlottesville, VA USA; 4 School of Population Health, Virginia Commonwealth University, Richmond, VA USA; 5 Department of Physical Medicine and Rehabilitation, Virginia Commonwealth University School of Medicine, Richmond, VA USA; 6 Department of Media and Information, Michigan State University, East Lansing, USA MI

**Correction** **:** **BMC Med 22, 243 (2024)**

**https://doi.org/10.1186/s12916-024-03465-1**

The original article [1] contained a typo in co-author, Raouf Gharbo’s name which has since been corrected.
